# Addressing Barriers to Enrolling Paid Caregivers in California: Systematic Review and Field Perspectives

**DOI:** 10.1093/geroni/igaf122.3877

**Published:** 2025-12-31

**Authors:** Aaron Luo

**Affiliations:** Yorba Linda High School, Yorba Linda, California, United States

## Abstract

Home care systems face persistent workforce shortages, with significant barriers to enrolling paid caregivers. This study examines barriers to the enrollment of paid home care workers, focusing on California’s regulatory and market contexts. A systematic literature review was conducted, supplemented by the perspective of practice from a field practitioner. Commonly identified barriers to enrolling paid home care workers include low wages, administrative burden, and limited career mobility. In California, state-specific enrollment requirements such as Home Care Aide (HCA) registration and Livescan background checks present additional challenges. These upfront expenses, often $70–$100 or more, reduced recruitment effectiveness by discouraging applicants and diminishing trust. Solutions identified vary based on who eventually pays for the costs, mainly including: (1) Agencies covering or paying back the upfront fees; (2) government help through subsidies or vouchers for those who qualify; (3) using workforce development funds to cover credentialing costs, and (4) making the application and background check process simpler and faster. Each has its own advantages and disadvantages. Pros and cons of each recommendation were discussed from the perspective of field practice. General recommendations include: implementing state-funded cost-offset programs for HCA registration and Live Scan; piloting expedited or mobile Live Scan services; broadening awareness campaigns in underserved communities; and creating public–private partnerships to cover credentialing costs. Addressing these barriers is critical to strengthening California’s paid home care workforce and providing essential services to older adults and those who rely on the services.

